# Physical–Chemical and Sensory Quality of Oat Milk Produced Using Different Cultivars

**DOI:** 10.3390/foods12061165

**Published:** 2023-03-09

**Authors:** Sumei Zhou, Qiuju Jia, Lulu Cui, Ying Dai, Ruoning Li, Jian Tang, Jing Lu

**Affiliations:** Beijing Advanced Innovation Center for Food Nutrition and Human Health, Beijing Key Laboratory of Flavor Chemistry, School of Food and Health, Beijing Technology and Business University, Beijing 100048, China

**Keywords:** oat cultivars, oat milk, nutritional composition, stability, sensory acceptability

## Abstract

Oat milk, as an emerging plant-based milk substitute, has become popular in recent years. However, the stability and flavor of oat milk products are hindering their quality. The examination of the processing capacities of potential oat cultivars could help to improve product quality. In the present study, the properties of oat milk produced from one Australian and three Chinese cultivars were compared. The stability of oat milk produced using our manufacturing process was superior to the commercial product and was highly influenced by cultivars. Positive correlations of the cultivar’s protein and plant cell debris content with the final products’ separation rate, and the cultivar’s lipid content with the final products’ creaming, were observed. Among the investigated cultivars, Chinese Bayou 01 (ZBY01) was the most suitable for oat milk processing. Oat milk produced with this cultivar has better stability and sensory acceptability. It can provide around 1% of protein, 9.84 mg/mL of β-glucan, and 70.96 mg GAE/100 g DW of polyphenols. Our results support one Chinese cultivar for oat milk processing and provide possible criteria for raw material selection.

## 1. Introduction

Oats rank fifth in the world’s cereal production, after corn, rice, wheat, and barley [1]. Oats are typically used as forage [2]. With the discovery of the beneficial effects of oats, around 14% of oat production is dedicated to the food industry nowadays [3]. Oats were first used as a food source by Germanic tribes in the first century, and they gradually gained popularity throughout the world [2]. Oats can be consumed as porridge, flour, or flakes. They are also used in bread and snack production as ingredients [4]. Oat-based beverages, such as oat milk, oat–berry beverages, and oat-based yogurt, are becoming more and more popular nowadays [4]. These beverages are ideal as a dairy substitute for people with celiac disease, milk allergies, or lactose intolerance.

In China, around 86% of northerners suffer from lactose malabsorption to various degrees [5], and the occurrence of lactose intolerance in Chinese children is 12.2% at age 3–5 years, 33.1% at age 7–8 years, and 30.5% at age 11–13 years, respectively [6]. Because a substantial proportion of Chinese individuals are lactose-intolerant, it is quite promising to develop plant-based milk products as dairy substitutes. A wide variety of plants are currently used to develop plant-based milk, such as soybeans, almonds, rice, coconut, oats, peas, etc., of which oat milk is a recently emerging product. Oat milk is high in β-glucan, and thus has potential benefits for hypercholesterolemia and hyperglycemia [7]. Meanwhile, the amino acid balance in oat proteins outperforms that of other cereal proteins [8]. The lipid content in oats is relatively low, with a high amount of unsaturated fatty acids [9]. The phytochemical compounds in oat milk may play an important role in its antioxidative activities [8]. Collectively, all these properties could indicate oat milk as a good dairy substitute for people who suffer from milk allergies and lactose intolerance.

Oats have been grown in China for more than 2000 years. Oats are widely consumed in Shan’xi and Inner Mongolia as flour [10]. The oat variety is mainly naked (Avena nuda) in China, differing from the husked one (Avena sativa) used in other countries [11], such as Australia, Russia, and Denmark. However, to our knowledge, no oat milk product is well consumed and widely accepted in the Chinese market, which may be due to the poor quality and taste of oat milk products. Among all the parameters that influence the quality and acceptability of oat milk, stability is the most important. The stability of oat milk could be influenced by the raw materials’ properties and processing procedures [12].

There are around 50 naked oat cultivars grown in China. These cultivars are different in terms of macronutrients and phytochemical composition [13], which could influence the quality of the oat milk. In the present study, three naked oat cultivars from China and one husked oat cultivar from Australia were used to produce oat milk to elucidate how the cultivars affected the oat milks’ properties. The influence of cultivars on oat milk quality and its possible reasons were comprehensively investigated. The results of the present study could guide the selection of suitable raw materials for oat milk production.

## 2. Materials and Methods

### 2.1. Materials

Three naked oat cultivars (Avena nuda, Bayou 01, Bayou 09, and Huazao 02) and one husked oat cultivar (Avena sativa) were used for oat milk production. Bayou 01 (ZBY 01) and Bayou 09 (ZBY 09) were obtained from the Zhangjiakou Academy of Agricultural Sciences (Zhangjiakou, China). Huazao 02 (ZHH02) was obtained from the Shanxi Academy of Agricultural Sciences (Taiyuan, China). Australian oats (AO) were obtained from SeaMild (Hezhou, China). Amylase (AF340362), glycosylase (AMG300L), and β-glucanase (CNNBC031) were purchased from Novozymes (Beijing, China) Biotechnology Co., Ltd.

### 2.2. Preparation of Oat Milk

**Figure 1:**
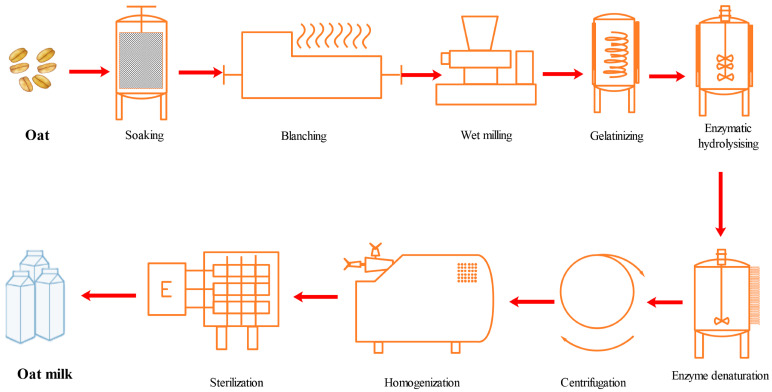
The manufacturing process of oat milk.

The manufacturing process is shown in Figure 1. Oats were blanched (95 °C, 5 min) after cleaning. Then, the samples were milled with six times as much water and kept at 95 °C for 10 min. Then, α-amylase (0.3 mL/100 g grains), glycosylase (0.5 mL/100 g grains), and β-glucanase (0.05 mL/100 g grains) were added to the samples. The samples were hydrolyzed at 100 °C for 1 h and then filtered through a 200-mesh cloth. Then, 1% of canola oil was added. The mixture was dispersed at 5000 rpm for 1 min, followed by two cycles of high-pressure homogenization at 45 MPa (Scientz-150, Ningbo Xinzhi Biotechnology Co., Ltd., Ningbo, China). Finally, the samples were sterilized for 5 s at 135 °C (HZ-SJJ, Shanghai Huizhan Experimental Equipment Co., Ltd., Shanghai, China).

### 2.3. Nutritional Composition Analysis

The nutritional composition analysis of cultivars and oat milk products was conducted as follows. The protein content was determined according to the Kjeldahl method [14]. The fat content was measured following the Soxhlet method [15]. Total starch [16] and amylose [17] content were determined by the glucose oxidase/peroxidase (GOPOD) method using an enzymatic assay kit (Megazyme International Ireland Ltd., Wicklow, Ireland). Ash [18] and moisture [19] content were determined according to the AOAC’s official method. The content of others was calculated as follows: other (%) = 100−rotein(%)−starch(%)−lipid(%)−ash(%)−water(%). The β-glucan concentration was measured using an assay kit for mixed linkage β-glucan [20] (Megazyme, Bray, Ireland). The polyphenol content was determined using Folin’s phenol and spectrometry [13]. In brief, 1 g of sample was weighed, dispersed in 80% ethanol, and extracted using an ultrasound treatment of 200 watts for 10 min. The extracts were mixed and fixed to 100 mL. Then, 0.2 mL sample, 0.8 mL 7.5% sodium carbonate, and 1 mL diluted Folin’s reagent were mixed and kept in the dark for 2 h. The absorbance was then measured at 765 nm, and the results were expressed as gallic acid equivalents (mg GAE per 100 g DW).

### 2.4. Physical–Chemical Properties of Products

#### 2.4.1. Particle Size Distribution

The particle sizes of oat milk were measured using a laser diffraction particle analyzer (BT-9300ST, Bettersize, Dandong, China). The samples were diluted in the instrument with ultrapure water until the laser obscuration value was in the range of 10–20%. The refractive indices of particles and dispersant were 1.57 and 1.33, respectively.

#### 2.4.2. Zeta Potential

The zeta potential of the samples was determined by measuring the direction and velocity of droplet movement in the applied electric field using a Zeta Sizer Nano-ZS90 (Malvern Instruments, Worcestershire, UK). All samples were diluted with ultrapure water at a ratio of 1:199 (*v*/*v*) and then placed into the sample cell. Before measurement, the samples were maintained for 1 min at 25 °C.

#### 2.4.3. Physical Stability

Stability was measured using an analytical centrifuge (LUMiSizer^®^, LUM GmbH, Berlin, Germany). The parameters used for the measurement were set as follows: rotational speed, 2500 rpm; time, 7650 s; temperature, 25 °C; time interval, 30 s. The separation rate was the slope in % h^−1^ when plotting the % of transmission over time with 0 as the intercept. The height of sediment and creaming was calculated for the last profile by subtracting the position with ≤20% light transmission from the bottom of the cell or meniscus, respectively [21]. The instability index characterized the separation of emulsions under accelerated gravitational force at a given time. It was between 0 and 1, where 0 represented the highest stability, indicating that there was no transmission or separation of the emulsion, and 1 represented the lowest stability, indicating the complete separation of phases [22].

#### 2.4.4. Apparent Viscosity

The rheological properties of oat milk were determined using a rheometer (MARS iQ Ai, Thermo, Carlsbad, CA, USA). The CC27 rotor was selected. The measured temperature was 25 °C. The viscosity of different samples was measured with a shear rate sweep test in the shear rate range of 0 to 100 s^−1^. The temperature was equilibrated for 3 min before measurement. The measurements were carried out in triplicate using the software. The power law model was used to evaluate the rheological properties of the samples:

(1) $$\tau =k\gamma^{n}\;$$

where *τ* is the shear stress, *k* is the consistency coefficient, *n* is the flow behavior index, and *γ* is the shear rate.

### 2.5. Sensory Evaluation

Oat milk produced from four cultivars was evaluated by 15 untrained panelists. All panelists were volunteers. Participants gave informed consent via the statement, “I am aware that my responses are confidential, and I agree to participate in this sensory evaluation”. They were able to withdraw from the survey at any time without giving a reason. The products tested were safe for consumption. All materials used during oat milk processing were food-grade and the product was sterilized (Section 2.2). The sensory attributes included color, aroma, mouthfeel, taste, and appearance of oat milk. Participants were asked to score samples from 1 to 10 on each attribute (Table 1).

### 2.6. Statistical Analysis

All experiments were performed in triplicate, and the results were expressed as the mean ± standard deviation (SD) in this study. One-way ANOVA was applied for significant difference analysis (*p* < 0.05). Pearson’s correlation was applied to investigate the relationships among the nutritional composition and stability of products.

## 3. Results and Discussion

### 3.1. Chemical Composition of Oat Cultivars

The protein content of these cultivars was between 11.85% and 12.98% (Table 2), which was similar to previous findings. The protein content of oats was between 10% and 20% [23,24,25]. There was no significant difference in protein content between naked and husked oats. ZBY09 contained 59.82% (*w*/*w*) of starch, which was the lowest among the four cultivars, and the starch content of ZBY01 was 66.63%, which was the highest value among different cultivars. The lipid content of AO was the highest (9.00%), and it was significantly different from that of the naked oat cultivars from China (4.23–6.59%). Of note, the fat content of the naked oats in the present study was significantly lower than that previously reported [23], while the fat content of the husked oats was comparable to that of dehulled oats reported previously [23].

### 3.2. Physical Properties of Oat Milk

**Figure 2:**
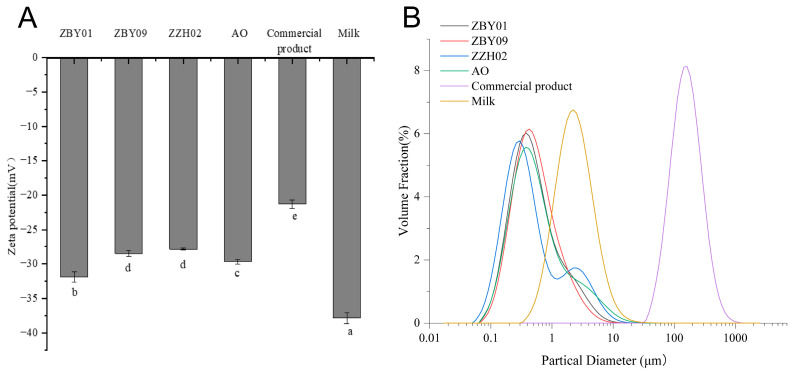
The ζ-potential (**A**) and particle size distribution (**B**) of commercial oat milk product, UHT bovine milk, and oat milk products produced from different cultivars. a,b,c,d,e, *p* < 0.05.

The ζ-potential in oat milk from various cultivars was investigated. The particles in the oat milk were negatively charged (Figure 2A). The absolute value of the ζ-potential of ZBY01 (−31.93 ± 0.74 mV) was significantly larger than that of ZBY09 (−28.50 ± 0.43 mV), ZZH02 (−27.87 ± 0.12 mV), and AO (−29.70 ± 0.29 mV). All absolute values of the ζ-potential of oat milk produced using our method were significantly higher than those of commercial product (−21.3 ± 0.61 mV), but lower than those of UHT milk (−37.9 ± 0.79 mV) (Figure 2A). The ζ-potential is used to assess the stability of a colloidal system. A ζ-potential of 30 to 40 mV is normally considered moderately stable, while a system with a ζ-potential of 20–30 mV is considered inherently unstable [26]. It seems that the oat milk produced from ZBY01 was more stable than other samples and comparable to UHT milk. Its absolute value was higher than that of the other plant-based drinks. Tangsuphoom et al. found that the ζ-potential of a coconut drink was −16 mV [27], and almond and hazelnut drinks had a ζ-potential of −18 mV and −22 mV, respectively [28].

The average particle size was also analyzed. Oat milk produced from ZZH02 had the lowest average particle size (0.43 ± 0.02 μm). The average particle size of the oat milk produced from ZBY01, ZBY09, and AO was 0.48 ± 0.004 μm, 0.48 ± 0.009 μm, and 0.54 ± 0.02 μm, respectively. These particle sizes were smaller than those of UHT milk (2.18 ± 0.02 μm). The particle size of the commercial oat milk was the largest (147.41 ± 4.001 μm). However, a bimodal distribution could be observed for the ZHH02 oat milk (Figure 2B). Large particles, with an average particle size of 2 μm, accounted for a certain portion. Although not obvious, a bimodal distribution could also be seen for the samples of ZBY01 and AO (Figure 2B). A bimodal distribution has been reported in plant-based drinks previously [29]. The larger particles could contribute to the sedimentation of the system [29]. However, in general, a smaller particle size and distribution are preferred to obtain stable plant-based beverages [30]. The density-based separation of oat milk is not only influenced by the particle size, but is also influenced by the gravity, density, and viscosity of the continuous phase [12].

**Figure 3:**
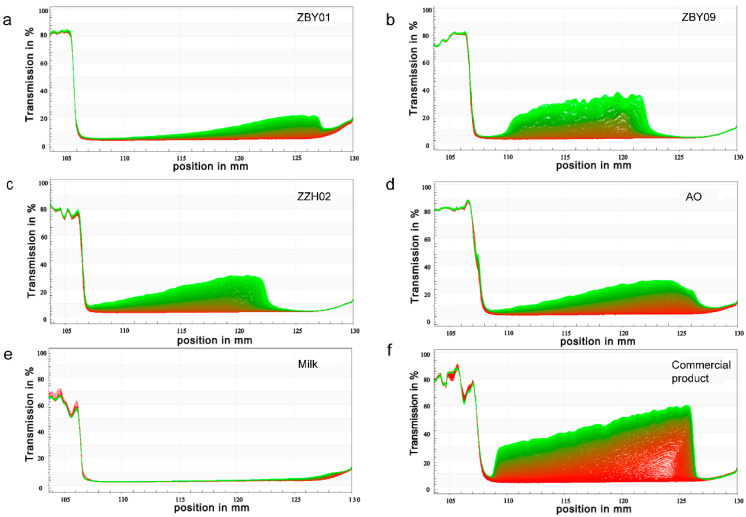
The stability of oat milk analyzed by using Lumisizer. (**a**), the stability of oat milk made of ZBY01; (**b**), the stability of oat milk made of ZBY09; (**c**), the stability of oat milk made of ZZH02; (**d**), the stability of oat milk made of AO; (**e**), the stability of UHT bovine milk; (**f**), the stability of commercial product. The red line in the bottom of each figure represents the first transmission record when the analysis started, the green line on the top of each figure represents the last transmission record during analysis.

The stability of the oat milk was also analyzed using a Lumisizer. The behavior of oat milks produced from different cultivars varied (Figure 3). The sedimentation of ZBY01 and AO was the lowest among all oat milk samples, while being higher than that of UHT bovine milk (Table 3, Figure 3). The sedimentation of ZZH02 was the highest among samples, which could be due to the large-sized particles (Figure 2B). Meanwhile, the creaming of ZZH02 was the lowest, and this could be linked to the composition of the raw materials. The reason for this will be discussed later. The creaming of all oat milks produced using our process was lower than that of commercial oat milk products (Table 3). Overall, the separation rate and instability index of ZBY01 were the lowest among the four cultivars. Moreover, the stability of the oat milk produced by us was better than that of the commercial one (Figure 3, Table 3). The UHT bovine milk was the most stable one, which was as expected [29] (Figure 3e, Table 3). All the oat milks produced by us and the commercial product showed pseudoplastic behavior (Table 4). The oat milk produced by us had a flow behavior index between 0.452 and 0.513 (Table 4) and a consistency index between 27.35 and 32.20 mPa s^n^, and they were not significantly different among samples. However, the flow behavior index and consistency index were significantly lower and higher than those of the commercial product, respectively (Table 4). In consistency, most of the plant-based products on the market also showed pseudoplastic behavior [31].

### 3.3. Correlation of Nutritional Composition of Cultivars and Physical Stability of Oat Milk

**Figure 4:**
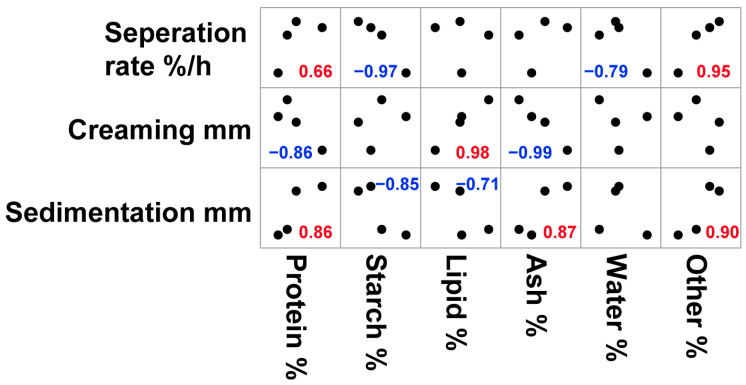
Correlation of nutritional composition of cultivars and physical stability of oat milk. The red number represents the positive correlation of the two parameters. The blue number represents the negative correlation of the two parameters.

The instability of the plant-based milk was mainly due to density-based separation, including creaming and sedimentation [12]. The Stoke–Einstein equation is normally used to explain this type of separation. The particle diameter, densities of the dispersed and continuous phase, gravity, and the viscosity of the continuous phase were the main factors that influenced the stability of the system [30]. These factors were mainly related to the processing procedure and the properties of the raw materials. The processing procedures were the same for all oat milk samples in the present study. Therefore, we speculated that the differences in stability could be due to the differences in oat cultivars. Thus, the correlation between the nutritional composition and separation rate, creaming, and the sedimentation of oat milk was analyzed. Since only four cultivars were utilized in the present study, no significance analysis was applied to the correlation analysis. Only correlation coefficients were dissected to explain the differences in physical stability. The main purpose of the correlation analysis in the present study was to identify the trend of change between the nutritional composition of raw materials and the physical stability of their products. Protein was highly correlated to the creaming and sedimentation of the sample, though it was not a linear correlation. The protein content, in particular, had a positive or negative correlation with the sedimentation or creaming level (Figure 4). Proteins could function as emulsifiers to wrap oil and provide steric stabilization on the lipid droplets to avoid their aggregation [12]. Thus, higher protein content could provide more emulsifiers to stabilize lipid droplets in the system [30], which leads to lower creaming. However, the emulsifying properties of proteins cannot be explained only by their content but also by their solubility, structure, and hydrophilicity/hydrophobicity [32]. We noticed that the protein content of ZBY01 was the lowest, while the creaming of this sample was not the highest, although the creaming was not significantly different among ZBY01, ZBY09, and AO (Table 3). This could be due to the superior amino acid composition, as the ratio of hydrophilic/hydrophobic amino acids was the highest in proteins from ZBY01 (Table S1). This could be a benefit of the increased emulsifying properties of ZBY01 proteins. On the other hand, proteins could be denatured during heat treatment [33], and aggregation was formed, which contributed to the sedimentation of the system. In oat milk, 70–80% of proteins were 12S globulins [25], and they were not significantly different among samples (Figure S1). Thus, the content rather than the composition of proteins could explain the correlation between proteins and sedimentation. The higher protein content led to more sedimentation. Overall, in the oat milk system of the present study, the lower protein content of the raw material was preferred for a lower separation rate of the whole system (Figure 4). The greatest component of oat cultivars was starch, which is correlated to the overall separation rate and sedimentation. The higher the content of starch, the lower the level of the separation rate and sedimentation. During processing, the starch of the raw material was partly hydrolyzed to dextrin and reducing sugar [34]. In the present study, the dextrose equivalent (DE) value of the oat milk was around 17%. The hydrolysis of starch led to a decrease in the viscosity of the system [35] and influenced the stability of the oat milk [12]. However, the viscosities of the oat milk produced from the four cultivars were not significantly different from each other (Table 4). Protein and starch are the two main components of oat kernels, and the effects of these two components were almost opposite to each other. Thus, we speculated that the influence of the starch might be due to its negative correlation with protein content. Lipid content is almost linearly correlated with creaming and negatively correlated with sedimentation. During processing, 1% of canola oil was added to the system, and the lipid fraction in the raw materials contributed almost half of the total lipids in the final product; thus, more lipids led to more creaming [12]. Other fractions, except for protein, starch, lipid, ash, and water, mainly including plant cell materials, were positively correlated with the separation rate and sedimentation. These components were mainly high-density materials, which led to the sedimentation of the system [12]. The higher the proportions of these components, the more sedimentation occurred. In the present study, ZBY09 and ZZH02 contained more plant cell material than the other two cultivars. Thus, the sedimentation of the oat milk produced from these two cultivars was higher than that of the other two samples. In the four cultivars studied in the present study, the oat milk produced from ZBY01 showed the best stability; it contained less protein, more starch, and less plant cell debris. It seems that oat materials with such characteristics could be preferred for stable oat milk production. Since this interpretation was based on four cultivars, more types of cultivars could be used for further investigation.

### 3.4. Sensory Evaluation of Oat Milk

**Figure 5:**
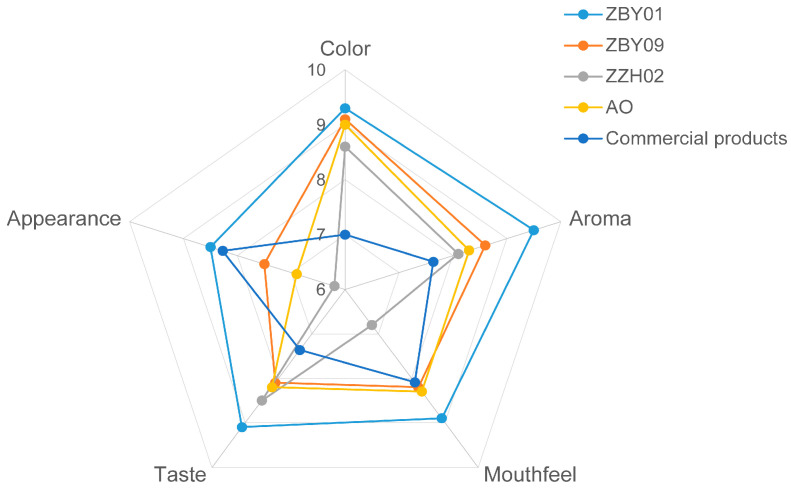
Sensory evaluation of oat milk.

The sensory evaluation was conducted by 15 untrained panelists. They were asked to evaluate the color, aroma, taste, mouthfeel, and appearance of the four oat milk samples by employing a 10-point grading scale. Oat milk produced with ZBY01 had the best color, aroma, taste, mouthfeel, and appearance (Figure 5). Meanwhile, oat milk produced from ZZH02 had the lowest values of color, aroma, mouthfeel, and appearance. Oat milk derived from ZBY09 and AO had similar sensory properties, except for the appearance (Figure 5). The differences in the appearance of these two samples could be due to the visible sedimentation of AO. The overall acceptability of ZBY01 was the highest among the four samples. In the sensory evaluation of oat milk, flavor and appearance could influence the overall acceptability of the product [36,37] and need to be improved during oat milk production. The oat milk produced by us had a superior flavor and a slightly better appearance compared to the commercial product (Figure 5).

### 3.5. Nutritional Composition of Oat Milk Samples

The nutritional composition of the four oat milk samples and the commercial product was analyzed. The protein content was not significantly different from each other. The oat milk obtained by our process can provide around 1% of protein (Table 5), which is comparable to or higher than previously reported commercial products (0.3–1.0% of protein) [38]. Moreover, it is not significantly different from the commercial product analyzed in the present study (Table 5). Notably, the protein content of ZBY01 was significantly lower than that of the other three cultivars, but the protein content of the final product was comparable to that of the other three samples. The lipid content was different among samples, which could be due to differences in the raw materials. The lipid content of oat milk produced from ZBY01, ZBY09, and AO was significantly higher, whereas the lipid content of the oat milk produced from ZZH02 was comparable to that of the commercial product and previously reported oat milk [38]. The oat milk samples and commercial product can provide around 60–73 mg/mL of carbohydrates, which is comparable to previous reports [38]. The carbohydrate content of oat milk produced from ZBY01 was the lowest and it was significantly lower than that of the commercial product. Besides the main nutrients, oat milk could also provide a certain amount of bioactive components, including β-glucan and polyphenols (Table 5). The content of β-glucan and polyphenols in the ZBY01 oat milk was the highest of the four cultivars, and the β-glucan and total polyphenol content of the oat milk produced by us were significantly higher than that of the commercial product. These constituents could help to improve the health benefits of oat milk products, such as their antioxidative activity and anti-hyperglycemia effects, etc. [8].

## 4. Conclusions

Three local Chinese cultivars and one from Australia were used to prepare oat milk products. The ZBY01 oat milk was the most stable and acceptable. It had the ζ-potential of −31.93 ± 0.74 mV, a size of 0.48 ± 0.004 μm on average, and a separation rate of 1.06 ± 0.05%/h. The flavor and appearance of the ZBY01 oat milk were better than those of the other three. Besides providing around 1% of protein, the ZBY01 oat milk also had the highest level of β-glucan and polyphenols of the four oat milk tested. The stability of oat milk could be related to the characterization of the raw materials. ZBY01 contained low protein content, high starch content, and low plant cell debris. Raw oat materials with compositions similar to ZBY01 might be preferred for oat milk production. However, because these criteria were based on four cultivars, more raw materials are needed for further rigorous studies.

## Figures and Tables

**Table 1:** Criteria for sensory evaluation of oat milk.

Index	Evaluation Standards	Score
Color	Uniform color, milky white	8–10
(10 points)	Slightly dull, nearly milky white	6–7
	Dull and tends to be gray	4–5
	Dull and not acceptable	1–3
Aroma	Oat aroma and strong	8–10
(10 points)	Oat aroma and not strong	6–7
	Little oat aroma, clear fishy smell	4–5
	No oat aroma, fishy smell and not acceptable	1–3
Mouthfeel	Smoothy and no graininess	8–10
(10 points)	Less smoothy, slightly graininess	6–7
	Not smoothy, more graininess	4–5
	Rough taste and serious graininess	1–3
Taste	Moderate sweetness, pleasant taste	8–10
(10 points)	Moderate sweetness, slightly bitter or oily	6–7
	Plain taste, bitter or oily	4–5
	Obvious bitter or oily taste, not acceptable	1–3
Appearance	Uniform system, no visible separation	8–10
(10 points)	Relatively uneven system, slight wall hanging or separation	6–7
	Non-uniform system, certain wall hanging phenomenon	4–5
	Unstable system, obvious separation	1–3

**Table 2:** Chemical composition of oat cultivars.

Chemical Composition, %	Cultivar			
	ZBY01	ZBY09	ZZH02	AO
Protein	11.85 ± 0.20 ^b^	12.98 ± 0.54 ^ab^	14.60 ± 0.68 ^a^	12.42 ± 0.68 ^ab^
Starch	66.63 ± 0.86 ^a^	59.82 ± 0.17 ^c^	61.61 ± 1.89 ^bc^	63.16 ± 0.52 ^b^
Amylose	37.09 ± 0.42 ^a^	36.34 ± 0.82 ^a^	38.73 ± 1.35 ^a^	36.41 ± 1.62 ^a^
Lipid	6.59 ± 0.31 ^b^	6.44 ± 0.39 ^b^	4.23 ± 0.12 ^c^	9.00 ± 0.44 ^a^
Ash	1.57 ± 0.05 ^bc^	1.76 ± 0.06 ^b^	2.08 ± 0.06 ^a^	1.39 ± 0.01 ^c^
Water	10.41 ± 0.20 ^a^	8.72 ± 0.02 ^b^	8.90 ± 0.08 ^b^	7.85 ± 0.10 ^c^
Other	2.95 ± 0.81 ^d^	10.28 ± 0.62 ^a^	8.58 ± 0.14 ^b^	6.18 ± 0.89 ^c^

^a,b,c,d^, *p* < 0.05.

**Table 3:** Stability of oat milk produced by different cultivars, commercial product, and UHT bovine milk.

Sample	Separation Rate (%/h)	Sediment (mm)	Creaming (mm)	Instability Index
ZBY01	1.06 ± 0.05 ^d^	2.55 ± 0.05 ^d^	1.65 ± 0.15 ^b^	0.092 ± 0.003 ^c^
ZBY09	2.16 ± 0.02 ^b^	4.90 ± 0.20 ^b^	1.60 ± 0.20 ^b^	0.185 ± 0.001 ^b^
ZZH02	2.03 ± 0.02 ^b^	5.15 ± 0.35 ^a^	1.35 ± 0.25 ^c^	0.177 ± 0.001 ^b^
AO	1.87 ± 0.05 ^c^	2.85 ± 0.05 ^d^	1.80 ± 0.00 ^b^	0.152 ± 0.010 ^b^
UHT bovine milk	0.16 ± 0.01 ^e^	0.60 ± 0.20 ^e^	1.05 ± 0.15 ^c^	0.012 ± 0.001 ^d^
Commercial product	5.74 ± 0.12 ^a^	3.75 ± 0.05 ^c^	2.05 ± 0.35 ^a^	0.384 ± 0.004 ^a^

^a,b,c,d,e^, *p* < 0.05.

**Table 4:** Rheology properties of oat milk produced from different cultivars.

Sample	Apparent Viscosity		
	Consistency Index k (mPa s^n^)	Flow Behavior Index n	R^2^
ZBY01	31.35 ± 0.50 ^a^	0.492 ± 0.020 ^a^	0.999
ZBY09	30.30 ± 10.47 ^a^	0.513 ± 0.089 ^a^	0.999
ZZH02	32.2 ± 2.82 ^a^	0.463 ± 0.026 ^a^	0.999
AO	27.35 ± 2.33 ^a^	0.452 ± 0.006 ^a^	0.999
Commercial products	6.75 ± 0.50 ^b^	0.843 ± 0.001 ^b^	0.990

^a,b^, *p* < 0.05.

**Table 5:** Nutritional composition of oat milk.

	Protein, %	Lipid, %	Carbohydrate, mg/mL	β-Glucan, mg/mL	Total Polyphenols, mg GAE/100 g DW
ZBY01	0.95 ± 0.01 ^a^	1.88 ± 0.01 ^b^	60.35 ± 0.30 ^d^	9.84 ± 0.44 ^a^	70.96 ± 0.15 ^a^
ZBY09	1.05 ± 0.05 ^a^	1.87 ± 0.02 ^b^	63.22 ± 0.45 ^c^	9.12 ± 0.68 ^b^	60.03 ± 0.92 ^c^
ZZH02	1.11 ± 0.01 ^a^	1.51 ± 0.03 ^c^	71.86 ± 0.71 ^a^	8.77 ± 0.40 ^c^	62.96 ± 0.77 ^b^
AO	1.09 ± 0.12 ^a^	2.01 ± 0.04 ^a^	73.47 ± 0.40 ^a^	8.09 ± 0.04 ^d^	60.79 ± 1.39 ^c^
Commercial product	1.01 ± 0.02 ^a^	1.52 ± 0.02 ^c^	66.15 ± 0.28 ^b^	4.69 ± 0.12 ^e^	47.40 ± 1.9 ^d^

^a,b,c,d,e^, *p* < 0.05. DW: dry weight.

## Data Availability

Data are contained within the article or supplementary material.

## Supplementary Materials

The following supporting information can be downloaded at: https://www.mdpi.com/article/10.3390/foods12061165/s1, Figure S1: SDS-PAGE of proteins in four cultivars; Table S1: The ratio of hydrophilic/hydrophobic amino acids in four oat cultivars.

## References

[1] Papageorgiou M., Skendi A., Galanakis C.M. (2018). 1-Introduction to cereal processing and by-products. Sustainable Recovery and Reutilization of Cereal Processing By-Products.

[2] Hareland G.A., Manthey F.A., Caballero B. (2003). OATS. Encyclopedia of Food Sciences and Nutrition.

[3] Price R.K., Welch R.W., Caballero B. (2013). Cereal Grains. Encyclopedia of Human Nutrition.

[4] Sontag-Strohm T., Lehtinen P., Kaukovirta-Norja A., Arendt E.K., Dal Bello F. (2008). 8-Oat products and their current status in the celiac diet. Gluten-Free Cereal Products and Beverages.

[5] Yongfa W., Yongshan Y., Jiujin X., Ruofu D., Flatz S.D., Kühnau W., Flatz G. (1984). Prevalence of primary adult lactose malabsorption in three populations of northern China. Hum. Genet..

[6] Yang Y., He M., CUl H., B1AN L., Wang Z. (2000). The prevalence of lactase deficiency and lactose intolerance in Chinese children of different ages. Chin. Med. J..

[7] Martínez-Villaluenga C., Peñas E. (2017). Health benefits of oat: Current evidence and molecular mechanisms. Curr. Opin. Food Sci..

[8] Sethi S., Tyagi S.K., Anurag R.K. (2016). Plant-based milk alternatives an emerging segment of functional beverages: A review. J. Food Sci. Technol..

[9] Ribeiro T.B., Voss G.B., Coelho M.C., Pintado M.E., Bhat R. (2022). Chapter 33-Food waste and by-product valorization as an integrated approach with zero waste: Future challenges. Future Foods.

[10] Hu X.-Z., Zheng J.-M., Li X.-P., Xu C., Zhao Q. (2014). Chemical composition and sensory characteristics of oat flakes: A comparative study of naked oat flakes from China and hulled oat flakes from western countries. J. Cereal Sci..

[11] Zwer P.K., Wrigley C., Corke H., Seetharaman K., Faubion J. (2016). Oats: Overview. Encyclopedia of Food Grains.

[12] Patra T., Rinnan Å., Olsen K. (2021). The physical stability of plant-based drinks and the analysis methods thereof. Food Hydrocoll..

[13] Tong L.-T., Liu L.-Y., Zhong K., Wang Y., Guo L.-N., Zhou S.-M. (2014). Effects of Cultivar on Phenolic Content and Antioxidant Activity of Naked Oat in China. J. Integr. Agric..

[14] ISO (2014). ISO 8968-1: 2014 (IDF 20-1: 2014) Milk and Milk Products: Determination of Nitrogen Content-Part 1: Kjeldahl Principle and Crude Protein Calculation.

[15] ISO (2009). 659: 2009, Oilseeds—Determination of Oil Content.

[16] International A. (2000). AACC Methods 76-13.01.

[17] Gibson T.S., Solah V.A., McCleary B.V. (1997). A Procedure to Measure Amylose in Cereal Starches and Flours with Concanavalin A. J. Cereal Sci..

[18] Delwiche S. (2003). Official Method 923.03.

[19] AOAC (2010). AOAC Official Method 925.10: Solids (Total) and Moisture in Flour.

[20] AACC (1999). International Method 32-23.01, β-Glucan Content of Barley and Oats—Rapid Enzymatic Procedure.

[21] Jeske S., Zannini E., Cronin M.F., Arendt E.K. (2018). Impact of protease and amylase treatment on proteins and the product quality of a quinoa-based milk substitute. Food Funct..

[22] Kori A.H., Mahesar S.A., Sherazi S.T.H., Khatri U.A., Laghari Z.H., Panhwar T. (2021). Effect of process parameters on emulsion stability and droplet size of pomegranate oil-in-water. Grasas Y Aceites.

[23] Biel W., Jacyno E., Kawęcka M. (2014). Chemical composition of hulled, dehulled and naked oat grains. S. Afr. J. Anim. Sci..

[24] Moisio T., Forssell P., Partanen R., Damerau A., Hill S.E. (2015). Reorganisation of starch, proteins and lipids in extrusion of oats. J. Cereal Sci..

[25] Kumar L., Sehrawat R., Kong Y. (2021). Oat proteins: A perspective on functional properties. Lwt.

[26] Kumar A., Dixit C.K., Nimesh S., Chandra R., Gupta N. (2017). 3-Methods for characterization of nanoparticles. Advances in Nanomedicine for the Delivery of Therapeutic Nucleic Acids.

[27] Tangsuphoom N., Coupland J.N. (2008). Effect of pH and ionic strength on the physicochemical properties of coconut milk emulsions. J. Food Sci..

[28] Bernat N., Cháfer M., Rodríguez-García J., Chiralt A., González-Martínez C. (2015). Effect of high pressure homogenisation and heat treatment on physical properties and stability of almond and hazelnut milks. LWT-Food Sci. Technol..

[29] Durand A., Franks G.V., Hosken R.W. (2003). Particle sizes and stability of UHT bovine, cereal and grain milks. Food Hydrocoll..

[30] McClements D.J., Newman E., McClements I.F. (2019). Plant-based Milks: A Review of the Science Underpinning Their Design, Fabrication, and Performance. Compr. Rev. Food Sci. Food Saf..

[31] Jeske S., Zannini E., Arendt E.K. (2017). Evaluation of Physicochemical and Glycaemic Properties of Commercial Plant-Based Milk Substitutes. Plant Foods Hum. Nutr..

[32] Farooq Z., Boye J.I., Tiwari B.K., Gowen A., McKenna B. (2011). 11-Novel food and industrial applications of pulse flours and fractions. Pulse Foods.

[33] Runyon J.R., Sunilkumar B.A., Nilsson L., Rascon A., Bergenståhl B. (2015). The effect of heat treatment on the soluble protein content of oats. J. Cereal Sci..

[34] Uthumporn U., Zaidul I.S.M., Karim A.A. (2010). Hydrolysis of granular starch at sub-gelatinization temperature using a mixture of amylolytic enzymes. Food Bioprod. Process..

[35] Deswal A., Deora N.S., Mishra H.N. (2014). Optimization of Enzymatic Production Process of Oat Milk Using Response Surface Methodology. Food Bioprocess Technol..

[36] Yao Y., He W., Cai X., Bekhit A.E.-D.A., Xu B. (2022). Sensory, physicochemical and rheological properties of plant-based milk alternatives made from soybean, peanut, adlay, adzuki bean, oat and buckwheat. Int. J. Food Sci. Technol..

[37] Moss R., Barker S., Falkeisen A., Gorman M., Knowles S., McSweeney M.B. (2022). An investigation into consumer perception and attitudes towards plant-based alternatives to milk. Food Res. Int..

[38] Pointke M., Albrecht E.H., Geburt K., Gerken M., Traulsen I., Pawelzik E. (2022). A Comparative Analysis of Plant-Based Milk Alternatives Part 1: Composition, Sensory, and Nutritional Value. Sustainability.

