# Supplementary material for: Physical–Chemical and Sensory Quality of Oat Milk Produced Using Different Cultivars
Source: Foods. 2023 Mar 9;12(6):1165. doi: 10.3390/foods12061165 (PMC10048011; doi:10.3390/foods12061165)
Supplement: Supplementary file 1 [file foods-12-01165-s001.zip › foods-2250844-supplementary/Table S1.pdf]

Table S1 The ratio of hydrophilic/hydrophobic amino acids in four oat cultivars

	ZBY01	ZBY09	ZZH02	AO
Hydrophilic/Hydrophobic amino acids, %	56.54 ± 0.8^{a}	54.85 ± 0.63^{ab}	53.5 ± 0.07^{b}	55.94 ± 0.65^{a}
