# Supplementary figures and images for: Physical–Chemical and Sensory Quality of Oat Milk Produced Using Different Cultivars
Source: Foods. 2023 Mar 9;12(6):1165. doi: 10.3390/foods12061165 (PMC10048011; doi:10.3390/foods12061165)

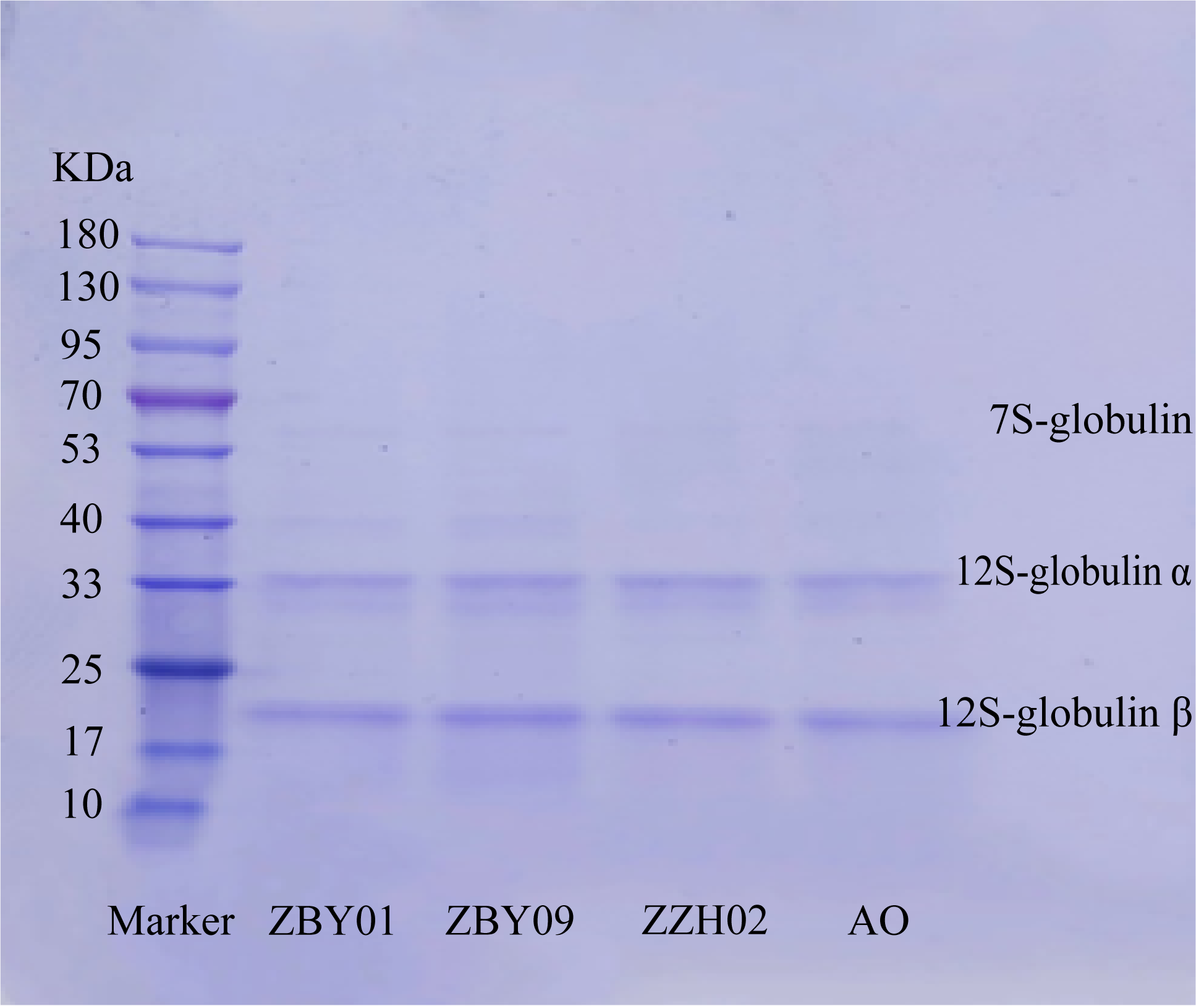

Supplement: Supplementary file 1 [file foods-12-01165-s001.zip › foods-2250844-supplementary/Figure S1.tif]
